# The Effect of Microfluidic Geometry on Myoblast Migration

**DOI:** 10.3390/mi10020143

**Published:** 2019-02-21

**Authors:** Rahul Atmaramani, Bryan J. Black, Kevin H. Lam, Vinit M. Sheth, Joseph J. Pancrazio, David W. Schmidtke, Nesreen Zoghoul Alsmadi

**Affiliations:** Department of Bioengineering, University of Texas at Dallas, Richardson, TX 75080, USA; rxa162330@utdallas.edu (R.A.); bjb140530@utdallas.edu (B.J.B.); Kevin.Lam2@utdallas.edu (K.H.L.); vms150130@utdallas.edu (V.M.S.); Joseph.Pancrazio@utdallas.edu (J.J.P.)

**Keywords:** microfluidics, myoblasts, migration, PDMS, microfabrication

## Abstract

In vitro systems comprised of wells interconnected by microchannels have emerged as a platform for the study of cell migration or multicellular models. In the present study, we systematically evaluated the effect of microchannel width on spontaneous myoblast migration across these microchannels—from the proximal to the distal chamber. Myoblast migration was examined in microfluidic devices with varying microchannel widths of 1.5–20 µm, and in chips with uniform microchannel widths over time spans that are relevant for myoblast-to-myofiber differentiation in vitro. We found that the likelihood of spontaneous myoblast migration was microchannel width dependent and that a width of 3 µm was necessary to limit spontaneous migration below 5% of cells in the seeded well after 48 h. These results inform the future design of Polydimethylsiloxane (PDMS) microchannel-based co-culture platforms as well as future in vitro studies of myoblast migration.

## 1. Introduction

Cell migration is integral to normal physiological function and also plays a role in pathological processes such as immune response [1], wound healing [2], and cancer metastasis [3]. In particular, myoblast cell motility has been studied extensively due to their involvement in the process of myogenesis, where these cells contact barriers in the form of connective tissue to form skeletal myofibers [4,5,6]. Migration assays used to investigate myoblast motility have mainly relied on two-dimensional surfaces, such as the wound-healing assay, which introduces a “wound” on a monolayer of cultured cells to test directed cell migration under influence of cell-matrix and cell-cell interactions [7]. However, these methods are limited to cell population analysis, have temporal limits, and preclude the incorporation of chemotactic gradients.

Microfluidic systems have increasingly been used as a platform for maintaining controlled microenvironments for the in vitro culture of complex cellular systems, which aim to recapitulate physiological conditions [8]. Myoblast migration and differentiation in microfluidic systems have been previously explored for mechanisms involved in disease states such as muscular dystrophy [9] and in the development of neuromuscular junctions in vitro [10,11,12,13]. In addition, the culture, alignment, and fusion of myoblasts is integral to the formation of skeletal myotubes in vitro and has been extensively studied in the development of engineered muscle tissue constructs using microfluidic chips [14,15]. In extension, differentiated myoblasts can be co-cultured with spinal motor neurons to examine the formation and maintenance of neuromuscular junctions [16] or co-cultured with different cells types (e.g., fibroblasts) to study the effects of soluble factor signaling mechanisms [17]. Surprisingly, limited work has been done to examine myoblast migration using microfluidic devices. To date, only one previous report has leveraged the use of microfluidic chambers to study cellular responses of primary human myoblast cells to chemoattractants [18], taking advantage of the stable establishment of gradients across chambers and their chronic maintenance via hydrostatic pressure. However, it fails to incorporate dimensional complexity, which aim to recapitulate cell responses in confined spaces. Prior evidence suggests that biophysical cues in the form of physical constraints influence myoblast cell proliferation, alignment, and fusion to form myotubes [19]. Therefore, insight on cellular migration behavior over a range of mechanisms is required to elucidate the complexity associated with the directed process of myogenesis.

Polydimethylsiloxane (PDMS)-based microfluidic microchannels have been used for the study of spontaneous migration under physical confinement of epithelial cells, tumor cell lines, and leukocytes [20,21,22,23]. However, the role of microchannel geometry on spontaneous myoblast migration has not been previously reported. Therefore, to take full advantage of this platform for either myoblast migration studies or the creation of multicellular models, it is important to understand how microchannel width influences myoblast behavior.

Here, we explore how the microchannel width influences myoblast migration by varying the widths of channels that connect the proximal (or cell seeding) chamber and the distal chamber. Studies performed in microfluidic chips that had a range of microchannel widths (1.5–20 µm), revealed width-dependent inhibition of myoblast migration into the distal chamber. Previous studies of myoblast migration in vivo using primary myoblast and mouse myoblast cell line (C2C12) transplanted into host tissue demonstrate special patterns of migration up to 48 h from site the of injection [24]. Therefore, further temporal analyses (24–48 h) were carried out to determine the ability of myoblasts to migrate across microchannel widths over time points relevant *in vivo*. As expected, we observed a width and length-dependent inhibition of myoblast transit through microchannels, with the lowest percentage of cells in the distal chamber observed at a 3 µm channel width and a length of 500 µm at 24 h. Our study provides insight into the maximum allowable channel widths necessary to retain myoblasts in separate chambers interconnected by microfluidic channels for rational design of co-culture systems and migration-based assays incorporating myoblasts.

## 2. Materials and Methods

### 2.1. Microfluidic Device Fabrication

To assess the effect of microfluidic geometry on myoblast migration, a complementary set of multilevel microfluidic devices were designed (Table 1). All microfluidic devices had two identical proximal and distal chambers with constant dimensions (1500 μm wide × 130 μm tall) that were connected by a series of parallel ladder microchannels that were orthogonal to the proximal and distal chambers. The spacing between ladder channels was either 50 or 60 μm, while the length of the connecting ladder channels was designed to be 100 or 500 μm. Depending on the device, the width of the ladder channels was kept constant (3, 4, 5 or 8 μm) or was variable (1.5–20 μm). A standard two-step photolithography technique was used to construct the multilevel photoresist templates utilized for generating the PDMS micro channels. In the first step a ~10 μm thick layer of KMPR 1005 negative photoresist (MicroChem, Westborough, MA, USA) was patterned on the silicon wafer (University Wafers, Boston, MA) to serve as the template for the connecting ladder channels. This layer was formed by spin coating (1500 RPM) 4ml of photoresist for 30 s, followed by baking at 100°C for 5 min, and then exposure to ultraviolet (UV) light at 335 mJ/cm^2^. After exposure, the wafer was post-baked for 2 min and then developed for 2 min using a SU—8 developer (MicroChem, Westborough, MA, USA). The wafer was then rinsed with 20 mL isopropyl alcohol (IPA) and dried under nitrogen stream.

In the second step, a ~130 μm thick layer of photoresist (KMPR 1050) was deposited by spin coating at 1300 RPM for 30 s followed by baking at 100°C for 20 min. The wafer was aligned and then exposed to UV light at 1831 mJ/cm^2^ to pattern the taller main channels (proximal/distal). The wafer was then baked at 100°C for 6 min, developed for 5 min using SU-8 developer, rinsed with 20 mL IPA, and dried under a stream of nitrogen.

Prior to pouring PDMS over the photoresist template, the wafer was first coated with 10 μL of (Tridecafluoro—1, 1, 2, 2—TetraHydrooctyl) Methyl dichlorosilane (Geleste Inc, Morrisville, PA, USA) by vapor deposition for 4 h under vacuum to improve the release of cured PDMS from the photoresist template.

The PDMS microfluidic devices were fabricated using previously described methods (Alsmadi et al. 2017). Briefly the curing agent and base were mixed in a 1:10 ratio, (Sylgard 184 Silicone Elastomer Kit; Dow Corning, Midland, MI, USA), poured over the photoresist template, and degassed for 1 h. The wafers then were placed in an oven at 80°C for at least 1 h to cure. The channels were then removed from the mold, and an outlet port was cut into the PDMS. The PDMS stamps were sealed to glass cover slips that were treated with a 28% solution of nitric acid for 1 h and then with a 1% AquaSil siliconizing solution for 15 s. PDMS stamps were permanently sealed to the glass coverslips by first treating the surface of the PDMS and the coverslip with plasma cleaner at high radio frequency (RF) for 1 min. To study myoblast migration in an open architecture without the presence of physical barriers, microfluidic devices were assembled using non-plasma bonding techniques adapted from Xona microfluidics (Temecula, CA, USA). Briefly, sterile 35 mm glass dishes were submerged in 50 µg/mL poly-D-lysine (PDL) over night at 37 °C. Following surface treatment, slides were washed three times in deionized (DI) water and allowed to dry. Previously fabricated and sterilized microfluidic devices were assembled onto the glass slide by placing the device directly above the glass and pressing the device until no unbound areas were present. Assembled devices were treated similarly as described in Section 2.2 and Section 2.3 with the exception that after cell adhesion, the microfluidic device was carefully removed via the use of forceps and 1.5 mL complete medium was added to the cells.

### 2.2. Microfluidic Device Preparation

Microfluidic devices were coated by the introduction of 10–20 μL of 50 µg/mL Poly-D-lysine (PDL) into the inlet ports of the proximal and distal chambers and incubated overnight at 37 °C. Following PDL coating, the devices were washed three times with sterile deionized water and subsequently treated with 20 µg/mL laminin for 1–2 h at 37 °C. Before introducing the cells into the devices, excess non-adherent laminin was removed from the inlet ports of both chambers and replaced with Hank’s balanced salt solution (Sigma Aldrich, St. Louis, MO, USA).

### 2.3. Primary Skeletal Myoblast Isolation and Culture

Primary skeletal myoblasts were isolated from adult mice (4–6 weeks old) as described previously with minor modifications [25]. Anesthetized adult male mice (ICR-CD1, Envigo RMS, Inc., Indianapolis, IN, USA) were euthanized by cervical dislocation in accordance with The University of Texas at Dallas Institutional Animal Care and Use Committee (IACUC) approved protocols. Briefly, the skin was removed from both hind limbs and the gastrocnemius and tibullus anterior muscles were surgically dissected, cleaned of tendons and fat, and cut into 3 mm × 3 mm sections. Dissected muscle sections were pooled and incubated in digestive buffer consisting of 2 mg/mL collagenase I (Sigma Aldrich, St. Louis, MO, USA) and 1% penicillin—streptomycin (PS) for 30 min at 37 °C. The tissue was mechanically triturated, filtered using a 40 µm sieve filter, pelleted via centrifugation, and resuspended in cell medium consisting of DMEM with GlutaMAX (Sigma Aldrich, St. Louis, MO, USA) supplemented with 10% fetal bovine serum and 1% PS. The resultant isolated satellite cells were cultured in cell medium in 150 cm^2^ CellBIND flasks (Sigma Aldrich, St. Louis, MO, USA) for 72 to 96 h at 37 °C and 10% CO_2_ until the cells reached 70% confluence.

Adherent myoblast cells were trypsinized and collected by centrifugation at 500 × g for 6 min and the resulting cell pellet was resuspended in fresh cell medium. The cells were concentrated to 90,000 viable cells in a 5 μL seeding volume, introduced into the proximal chamber inlet port of previously prepared microfluidic devices, and incubated for 30 min to 1 h to allow cell adhesion. Then, 50 μL of cell medium was added carefully to all inlet ports to prevent flow, which might cause detachment of cells. The cells were routinely maintained in medium for 24 or 48 h before imaging sessions. For all cases, the addition of cells and reagents and/or cell medium were carried out manually via pipetting at the entrance of inlet ports.

### 2.4. Cell Viability Assay

To determine the cell viability of cultured primary myoblasts in microfluidic devices a cell viability assay was performed using the LIVE/DEAD cytotoxicity kit for mammalian cells according to manufacturer’s protocol (Thermofisher, L3324, Waltham, MA, USA). Briefly, 90,000 cells were seeded in either microfluidic devices or glass bottom 24 well plates (control) and maintained for 48 h. Calcein AM and ethidium homodimer were reconstituted in DMEM with GlutaMAX at 2 µM and 4 µM, respectively. Fluorescent imaging was performed using a 10X objective on an inverted microscope (Nikon, Japan). Image analysis and cell counts were performed in ImageJ (NIH, Bethesda, MD, USA).

### 2.5. Growth Factor Gradient Generation for Chemotaxis Experiment

For chemotaxis experiments, 100 µL of cell medium was introduced into the proximal chamber where myoblasts were seeded, while 100 µL cell medium supplemented with 11 ng/mL HGF or 11 ng/mL BFGF or 10 μM FITC-conjugated dextran was introduced in to the distal chamber. For growth factor experiments, after a 24 h period, 2 µM Calcein AM was introduced in both chambers and cells were imaged as described below. For the visualization of the gradient generated by FITC molecules, images were acquired of both chambers every 30 min over an 8 h period.

### 2.6. Live Imaging Using Calcein AM

Calcein AM (Molecular Probes, Invitrogen, Waltham, MA, USA) was diluted to a 2 µM concentration in DMEM with GlutaMAX and 1% PS, vortexed, and warmed to 37 °C. Cell medium was removed from the cell inlet ports and replaced with 100 µl DMEM + Calcein AM. The cells were incubated for 10–15 min at 37 °C and 10% CO_2_. After incubation, the microfluidic device was placed on the microscope inside a humidified chamber and fluorescently imaged with an excitation of 494 nm and an emission of 571 nm. Large stitched images (4 × 4 fields) of labeled cells were acquired using a 10X objective to capture the proximal and distal chambers. Cell counts were performed in ImageJ and plotted using OriginPro software (OriginLab Corporation, Northampton, MA, USA) as described below.

### 2.7. Fluorescence Microscopy and Image Analysis

All epifluorescence and bright field imaging were carried out using an inverted microscope (Nikon, Japan) and all epifluorescent images were acquired using epifluorescent light sources (Lumnecor, Beaverton, OR, USA). During live imaging sessions of myoblasts stained with Calcein AM dye, the cells were maintained in a humidified chamber at 37 °C and 10% CO_2_ (OKOLAB USA Inc., San Francisco, CA, USA). All images were analyzed via automated cell counts performed using custom macros in ImageJ v.1.6 (NIH, Bethesda, MD, USA) as previously described with minor modifications [26]. Briefly, large stitched images were cropped into proximal and distal chamber components with a region of interest (ROI) of area 4 mm × 1500 µm, which spanned the length of the microchannels and the width of the chamber respectively. Images were auto-contrasted, and a Gaussian blur of sigma radius 2 was applied to smooth local intensity peaks, followed by an automated detection of local intensity maxima with a noise tolerance of 70, detected separately in both proximal and distal components. For multi-width microchannel devices, ROI spanning the microchannel widths (6 repeats per width) were used to segment the image and automated cell counts were performed as described above or mean fluorescence intensities were detected in whole segments in both proximal and distal chambers. To account for cells spanning at the boundary of two ROI’s (or two different microchannel width sections), cells were included in both sections. For the quantification and visualization of the gradient generation due to the diffusion of FITC through the microchannels, three sections spanning the length of all microchannels were acquired every 30 min for a total duration of 8 h. Individual images were stitched in ImageJ using the 2D stitching plugin. Line intensity profiles were acquired across microchannels and were defined from the boundary of the proximal to distal chamber with a total length of 100 μm. Data were plotted using OriginPro software (OriginLab Corporation, Northampton, MA, USA). The percentage migration was calculated as the ratio of the number of cells in distal chamber to the number of cells in the proximal chamber where cells were initially seeded.

### 2.8. Statistical Analysis

All statistical analysis was performed in Origin Pro 2017 (OriginLab Corp., Northampton, MA, USA). The comparison between groups was carried out using a two-sample t-test. To compare between group effects, a one-way ANOVA was utilized. In all cases, p < 0.05 was considered statistically significant. All data presented are expressed as mean ± standard error the mean (SEM).

## 3. Results

### 3.1. Myoblasts Migrate Spontaneously Across Microchannels

**Figure 1:**
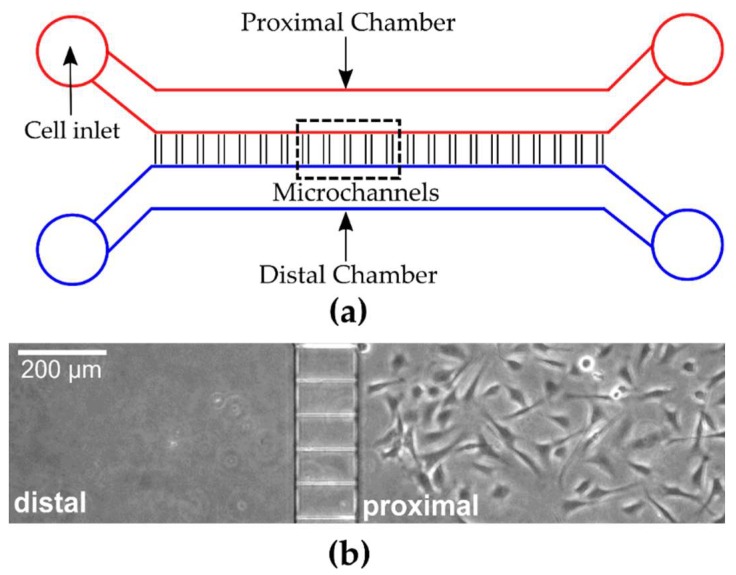
Schematic illustration of microfluidic device. (**a**) Representative drawing of PDMS based microfluidic device with distinct proximal and distal chambers. The red region indicates the proximal chamber in which the cells are seeded and is connected to a distal chamber (blue region) by an array of microchannels. (**b**) Phase contrast image of a section of the proximal and distal chamber after myoblast seeding in the proximal chamber.

**Figure 2:**
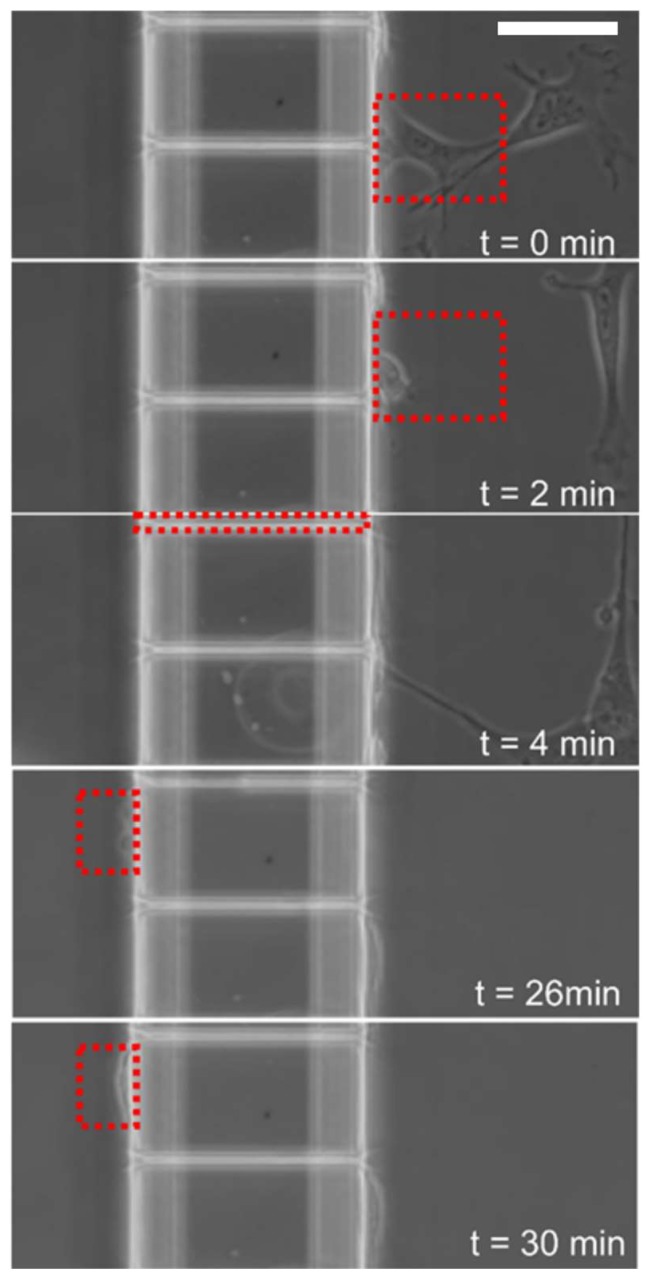
Time lapse microscopy revealed spontaneous migration of primary myoblast cells across PDMS based microfluidic channels. Phase contrast images of a representative cell at different time intervals traversing a microchannel of 5 μm width and 100 μm length. Red boxes indicate tracking of representative cell from the proximal (right) to distal chamber (left). Horizontal scale bar represents 50 μm.

To assess spontaneous myoblast migration across microchannels, PDMS-based microfluidic devices were fabricated which offer distinct chambers connected by an array of orthogonal ladder microchannels (Figure 1a). For the purpose of our study, the chamber where cells were seeded was denoted as the proximal chamber and the opposite chamber was denoted as the distal chamber (Figure 1a). The microchannels, which can act as barriers to free cellular movement between the proximal and distal chambers, were fabricated with varying lengths and widths. Cells were visualized in both chambers using fluorescence microscopy to quantify cell populations over time. With both chambers containing the same media, myoblasts were seeded through inlets in the proximal chamber to allow cells to initially adhere to the underlying substrate in the proximal chamber. In all of the experiments, the distal chamber was initially devoid of cells (Figure 1b). The cell viability of primary myoblasts cultured in microfluidic devices or open environments was assessed after 48 h using live/dead staining. We found a 91.6 ± 1.7% (mean ± STD, n = 3) cell viability in the microfluidic devices compared to 94.8 ± 2.3% (mean ± STD, n = 3) on glass substrates. The data suggests that no adverse effects were found on the growth and maintenance of cultures over 48 h in microfluidic devices In order to evaluate myoblast cell migration in the absence of microchannels, we carried out similar studies wherein the PDMS microchannel device was removed following cell adhesion. We found that 29.4 ± 2.3% (mean ± STD, n = 2) of the seeded cells migrated across the same 100 µm distance in the absence of microchannel confinement. Conversely, initial assessment of myoblast behavior after plating in devices with a microchannel width of 5 µm (length = 100 µm) revealed the movement of myoblasts from the proximal chamber through the ladder channels into the distal chamber. Figure 2 is a representative example of a single cell traversing completely from the proximal to the distal chamber over time periods which ranged from 30 min to 6 h. Consistent with prior observations of cell movement through PDMS based microchannels [22], we observed initial cell protrusions at the leading edge, complete elongation within the microchannels, and subsequent return to normal cellular shape and morphology in the distal chamber (Figure 2). These results demonstrate that myoblasts can be cultured in our microfluidic devices, and that myoblasts will migrate through the connecting ladder channels independent of an external concentration gradient.

### 3.2. Myoblast Migration Behavior is Dependent on the Width of Microchannels

**Figure 3:**
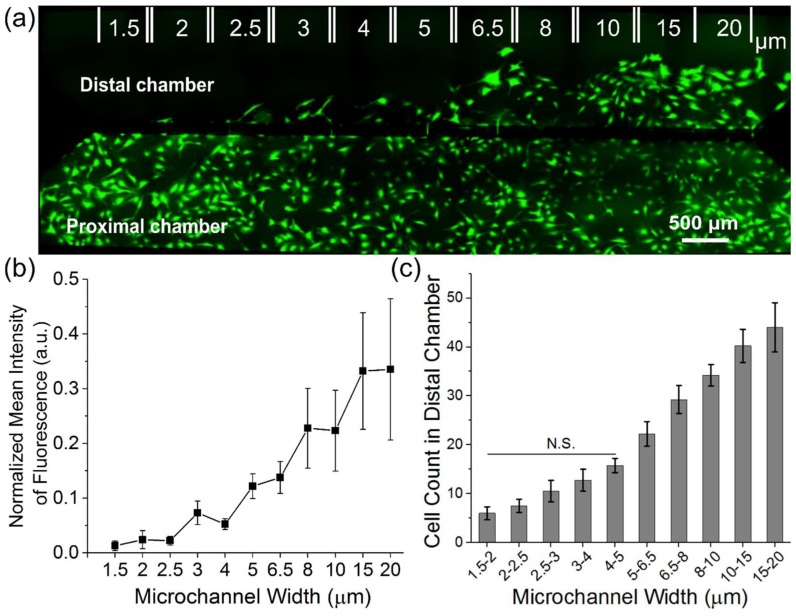
Migration behavior of primary myoblasts is dependent on the width of microchannels. (**a**) Representative fluorescent image of cells stained with Calcein AM in the proximal and distal chamber 48 h after seeding. The device consists of microchannel length 100 µm and variable widths (1.5–20 µm) with 6 repeating microchannels per width. (**b**) Normalized mean intensity of fluorescence in the distal chamber taken for each width from 1.5–20 µm sections. The mean intensity of fluorescence was normalized to the proximal chamber. (**c**) Quantification of the number of cells in the distal chamber after 48 h from 1.5–20 µm sections. To determine statistically significant differences in the cell count in the distal chamber as a function of microchannel width, a one-way ANOVA with a post hoc Tukey test was performed. Error bars represent standard error of the mean (SEM).

We next explored how myoblast migration is influenced by the width of the ladder microchannels. A multi-width microchannel device, with widths ranging from 1.5–20 µm and a length of 100 µm between proximal and distal chambers, was fabricated. Primary myoblasts were seeded in the proximal chamber and monitored for 48 h after which the cells in both chambers were stained with Calcein AM and visualized using fluorescence microscopy (Figure 3a). We observed microchannel width-dependent migration into the distal chamber. In regions where the proximal and distal chambers were separated by 20 µm wide microchannels there was little to no observable difference in cell density between proximal and distal chambers (as measured by the normalized mean intensity of fluorescence). In contrast, there were marked reductions in cell density in the distal chamber in the 1.5–3 µm sections (Figure 3b). To quantify the number of cell crossings, automated cell counts were performed in areas which spanned six identical repeats of a given microchannel width. As with the normalized mean fluorescence intensity, we observed a statistically significant difference in the cell count in the distal chamber as a function of microchannel width as determined by a one-way ANOVA (F (9, 30) = 20.368, p = 1.85 × 10^-10^). A post hoc tukey test revealed an increase in the number of crossings associated with 15–20 μm-wide channels (44 ± 5 cells, N = 4) as compared to 4–5 µm-wide microchannels (15 ± 1 cells, N = 4 devices, p < 0.001). However, there was no significant difference between the number of cell crossings between 15–20 µm-wide channels and 8–10 μm-wide microchannel widths (34 ± 2 cells, N = 4 devices, p = 0.5). These results suggest that there is a critical width/cross-section that restricts myoblast migration.

### 3.3. Temporal Assessment of Myoblast Migration Across 3, 4, 5 and 8 µm Width Microchannels

**Figure 4:**
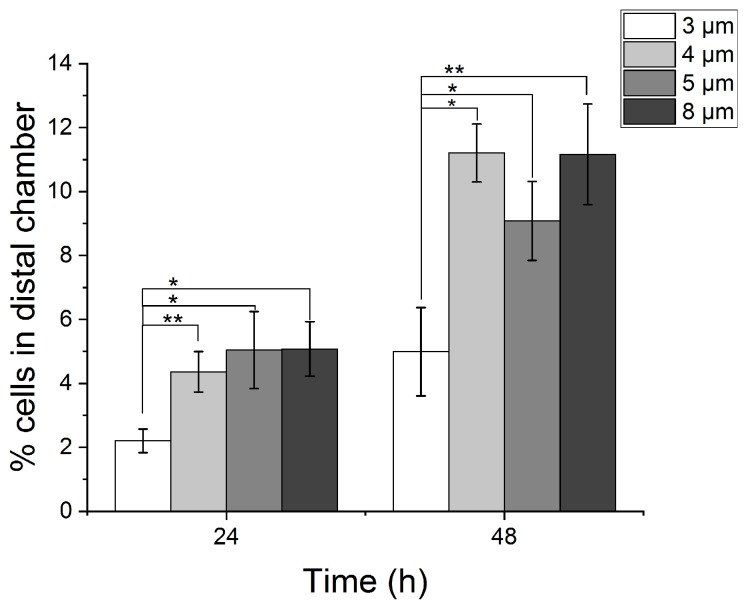
Assessment of primary myoblast migration over 24 and 48 h across microchannel widths of 3, 4, 5 and 8 µm and consistent length of 100 µm. The percentage of cells in the distal chamber was calculated as the ratio of number cells in the distal chamber to proximal chamber. Black asterisks are used to denote statistically significant differences (*p < 0.05, **p< 0.01) and error bars represent standard error of the mean (SEM).

Previous studies have demonstrated that myoblasts can migrate over long time scales, at the order of 1 min to 16 h, in response to growth factors, displaying chemokinesis, chemotaxis, and chemo-proliferation, processes which may occur from hours to days [18]. Therefore, we examined the time-dependence on the migration behavior of primary myoblasts across microchannels at 24 and 48 h time points in our preparation. We fabricated microfluidic devices with microchannels of uniform widths of either 3, 4, 5 or 8 µm but with a fixed length of 100 µm. After 24 h, only 2 ± 0.4% (N = 8 devices) of myoblasts were able to traverse the 3 µm microchannels, whereas 4 ± 0.6, 5 ± 1.2, and 5 ± 0.9 % of myoblasts were able to traverse the 4, 5 and 8 µm channels at the same time point (Figure 4). No significant difference was observed between the myoblast migration percentage between 4, 5 and 8 µm channels (p = 0.9). Similar results were observed following 48 h in culture, with the 3 µm channel width allowing the lowest percentage migration in comparison to the 4, 5 and 8 µm (Figure 4). Therefore, the effect of microchannel width on myoblast migration into the distal chamber is relatively conserved over time and small changes in width can have a significant effect on the number of cells traversing the microchannels.

### 3.4. Myoblast Migration Depends on the Length of Microchannels

**Figure 5:**
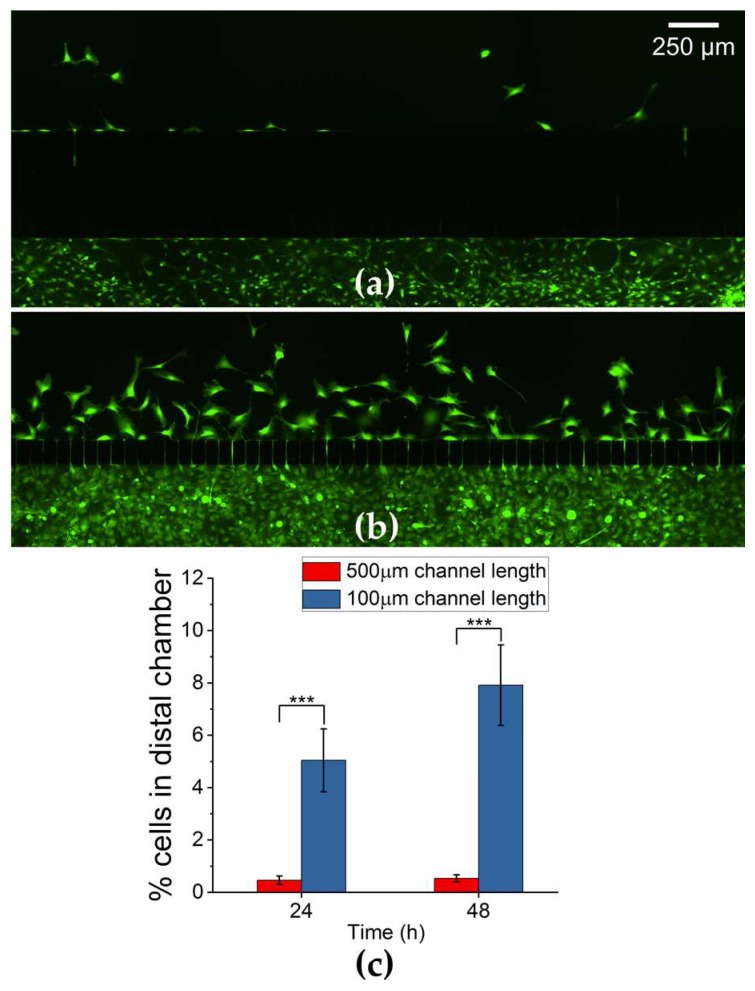
Primary myoblast cells subjected to microchannel lengths of 100 µm and 500 µm (width = 5 µm) exhibit length dependent constriction across microchannels. (**a**) Representative fluorescent image of myoblast cells across 500 µm microchannel length after 24 h. (**b**) Representative fluorescent image of myoblast cells in the across 100 µm microchannel length. (**c**) Plot of the percentage cells in distal chamber after 24 and 48 h of migration in devices with microchannel length 100 µm and 500 µm. Black asterisks are used to denote statistically significant differences (*p < 0.05, **p < 0.01, ***p < 0.001) and error bars represent standard error of the mean (SEM).

**Figure 6:**
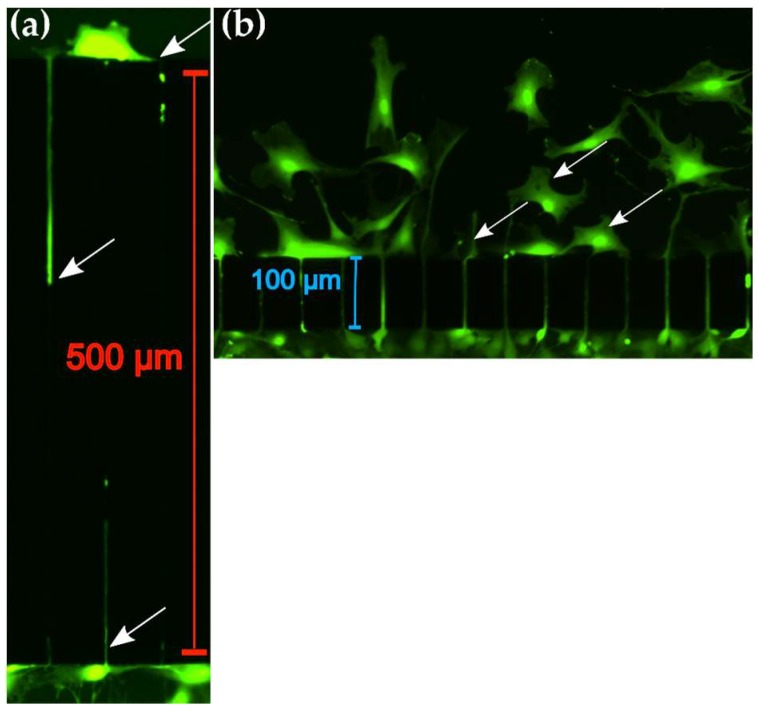
Effect of microchannel length on primary myoblast migration behavior. (**a**) Representative fluorescent image of cells crossing 500 μm length (width = 5 μm) microchannel from the proximal (bottom) to distal chamber (top). Arrows indicate cytoplasmic structures of cells in the microchannels. (**b**) Representative fluorescent image of cells crossing 100 μm length microchannel (width = 5 μm) from proximal (bottom) to distal (top) chamber. Arrows indicate cytoplasm structures after release of confinement from the microchannels.

To investigate the effects of microchannel length on myoblast migration, we quantified cell counts between chambers separated by microchannel lengths of 100 µm and 500 µm with a fixed width of 5 µm over a 24 and 48 h period. The 500 μm channel length reduced the number of myoblasts in the distal chamber significantly to only 0.4 ± 0.2% (N = 4 devices, p < 0.001) and 0.5 ± 0.1% (N = 5 devices, P < 0.001) at the 24 h and 48 h time points respectively (Figure 5a,c). In contrast, the 100 μm channel length allowed 5 ± 1.2% (N = 5 devices) and 8 ± 1.5% (N = 10 devices) at the 24 and 48 h time points respectively (Figure 5b,c). To traverse microchannels of length 500 μm, cells appeared to undergo deformations in the cytoplasmic structure within the microchannels. However, during transit, cells did not appear to fully extend across the 500 μm microchannel length (Figure 6a), suggesting that the migration through channels is enhanced when a portion of the cytoplasmic structure is relieved of confinement [27]. This was readily observed for myoblasts crossing the 100 μm microchannels, as they appeared to deform such that the entire length of the cell extended across the channels (Figure 6b). These results suggest that the migration dynamics of primary myoblasts are substantially influenced by the length of the microchannels.

### 3.5. Chemotactic Myoblast Migration Across Microchannels is Modulated in Response to Muscle-Specific Growth Factors

**Figure 7:**
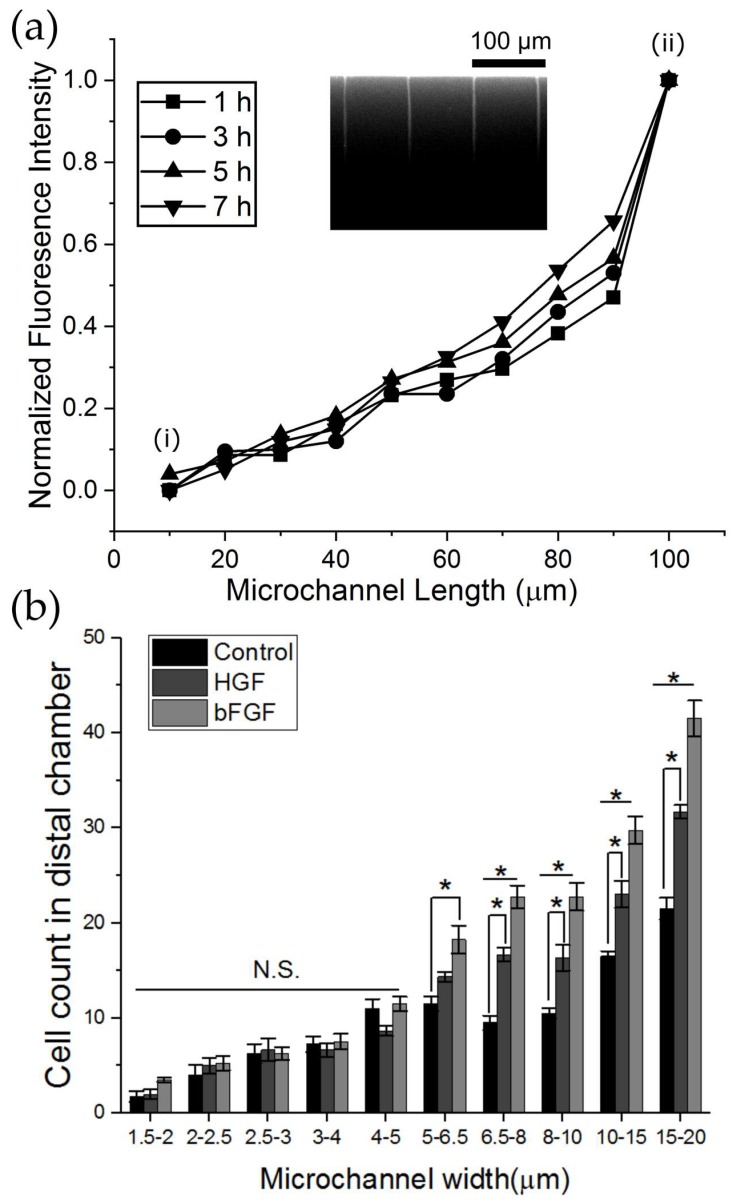
Effect of bFGF and HGF on myoblast migration across microchannels. (**a**) Normalized mean fluorescence intensity of FITC-conjugated dextran (molecular weight = 40,000 kDA) as a function of microchannel length at different time points. (i) Denotes the boundary of the proximal chamber or cell seeding chamber. (ii) Denotes the boundary of the distal chamber or migration chamber where FITC was introduced. Inset displays a representative image of 4 microchannels (width = 1.5 μm) at the 4 h time point which demonstrates the generation of a gradient of FITC molecules from distal (top) to proximal chamber (bottom). (**b**)The multi-width device (1.5–20 μm) with constant length of 100 µm was used to assess myoblast migration across microchannels in response to a chemoattractive gradient induced by either 11 ng/mL HGF or 11 ng/mL bFGF introduced into the distal chamber. Cell counts are expressed as the number of cell crossings into the distal chamber across binned channel widths. Each bin consists of 6 microchannels per width for a total of 12 microchannels. To determine statistically significant differences in the cell count between growth factor treatment and control for a given microchannel size range a one-way ANOVA with a post hoc Tukey test was performed. Black asterisks are used to denote statistically significant differences (*p < 0.05) and error bars represent standard error of the mean (SEM).

**Figure A1:**
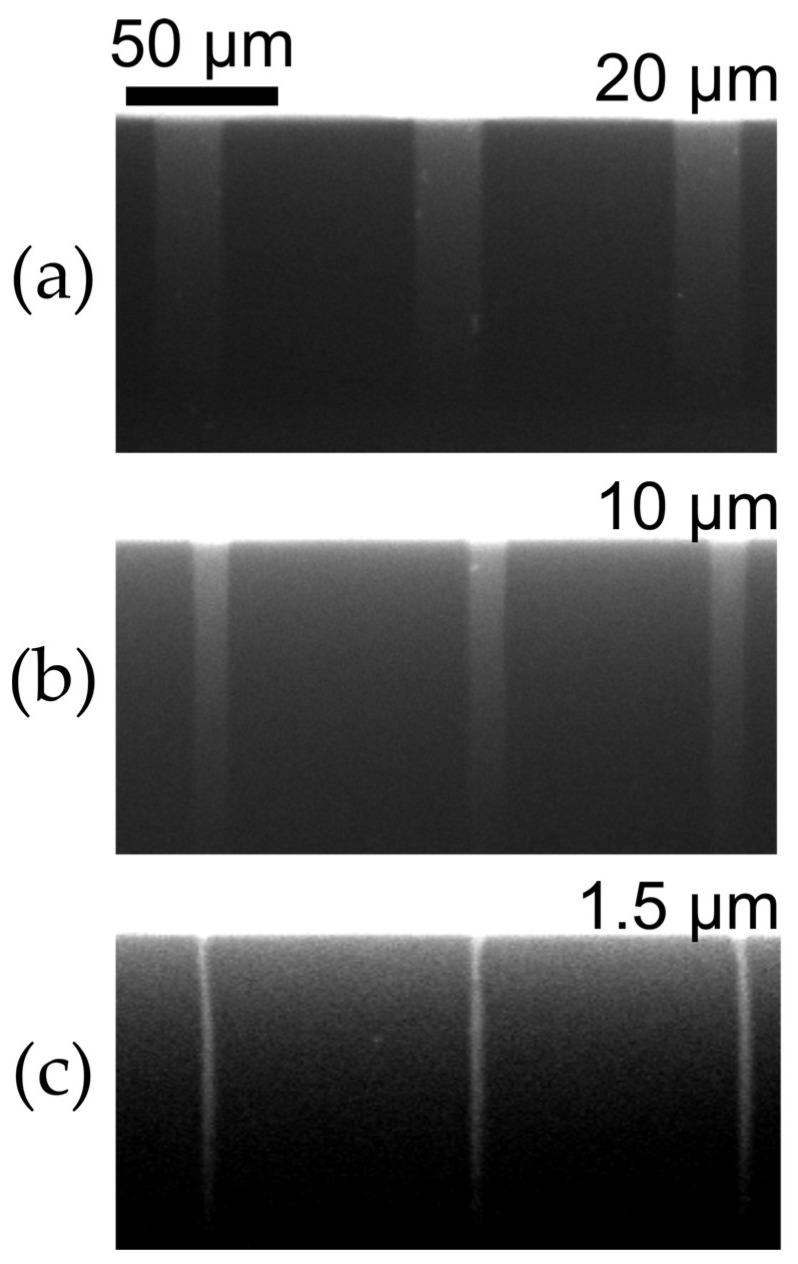
Representative images of FITC-conjugated dextran diffusion profile across microchannels of different widths at the 4 h time point. (**a**) 20 μm width microchannels. (**b**) 10 μm width microchannels. (**c**) 1.5 μm width microchannels.

To assess whether myoblast migration under confinement is enhanced in response to a chemoattractive gradient, we investigated the number of successful cell crossings into the distal chamber in response to hepatocyte growth factor (HGF) and basic fibroblast growth factor (bFGF) across microchannels of different widths. It has been previously described that both HGF and bFGF act as signaling growth factors during early stages of muscle development and muscle repair to guide migration of myogenic precursor cells [28,29]. Therefore, these muscle-specific growth factors are suitable to investigate growth factor mediated chemotaxis of myoblast cells. To characterize the temporal presence of the chemotactic field across the microchannels, FITC-conjugated dextran (molecular weight = 40,000 kDA) was used as a growth factor mimetic. To quantify the diffusion process, 10 µM FITC was introduced into the distal chamber and the proximal chamber was maintained in cell medium to model whether a concentration gradient would be established across the microchannels into the proximal chamber where cells are maintained. The gradient generated was visualized as fluorescence intensity profiles due to diffusion of FITC across the microchannels. The FITC molecules diffused into the proximal chamber immediately after introduction into the inlet ports and a linear gradient was established in approximately 1 h and was maintained for at least 8 h (Figure 7a). Interestingly, we observed a differential gradient dynamic as a function of microchannel width. For microchannels of 20 µm width we observed an exponential gradient and microchannels less than 3 µm we observed a nearly linear gradient (Figure A1). Similar observations have been made for straight linear microchannels of width 20 µm and is dependent on the permittivity allocated by wider dimensions that allow faster diffusion [30]. Myoblasts were seeded in the proximal chamber of multi-width microfluidic devices as described previously and cultured with cell medium, whereas, the distal chamber received cell medium supplemented with either 11 ng/mL HGF or 11 ng/mL bFGF and cells were allowed to migrate over a 24 h period. We observed significant increases in the number of cell crossings into the distal chamber in response to both HGF and bFGF between 15–20 µm-wide and 5–6.5 µm-wide microchannels as compared to control (Figure 7b). Furthermore, we also observed width-dependent reduction in chemotactic behavior. In the case of HGF treatment, at the 15–20 µm-wide microchannels, 31 ± 0.7 (N = 4) cells exited the microchannels compared to 14.3 ± 0.5 cells (N = 4) at the 5–6.5 µm-wide channels. Similar trends were observed for bFGF. Below the 5 µm width microchannels no significant difference between treatment groups and control was observed, indicating the presence of a critical width beyond which chemotactic behavior of myoblasts is restricted. Interestingly, we also observed significant differences in the chemotactic potential of bFGF and HGF, with the former inducing more cell migration between the 15–20 µm-wide and 5–6.5 µm-wide microchannels. In total, the results suggest both bFGF and HGF act as chemotactic factors for myoblast migration and this behavior is dependent on the width of microchannels.

## 4. Discussion

The objective of this study was to explore how microchannel width influences myoblast migration in a PDMS microfluidic structure. The PDMS-based structure affixed to a cover glass allowed for direct fluorescence imaging of both proximal and distal chambers and enabled visualization of population-level cellular migration behavior. Furthermore, in the absence of an externally applied concentration gradient of growth factors to influence migratory behavior, we observed spontaneous migration across the microchannels.

Our results revealed a width-dependent restriction to myoblast movement into the distal chamber, which was most pronounced in 3 to 8 µm wide channel. Over time spans of 24 to 48 h we observed the least number of cell crossings in the distal chamber in 3 µm channel width, with a two-fold increase for a 4 µm width. More importantly, similar trends were observed at both time points, although the net movement of cells over 48 h was higher as cells were allowed to migrate for longer periods.

Cell motility and migration behavior in confined spaces modeled by PDMS microchannels of varying geometric dimensions have been previously studied. For example, Tong et al. (2012) reported migration of HOS cells across microchannel widths between 3 μm to 50 μm. Cells in the 20 μm microchannel widths did not undergo deformation owing to the absence of constriction from the PDMS walls and had similar morphological characteristics as is observed on two-dimensional surfaces. However, cells within the 3 μm microchannels underwent the greatest deformation and had the lowest cell migration velocity compared to the 20 μm channel width [23]. In contrast to the present study, no significant difference was found for varying microchannel length (100 μm to 400 μm) on HOS migration velocity, which may be explained using a stable chemotactic gradient in these microfluidic structures to influence unidirectional and persistent migration. Similar results have been reported for the migration behavior of human pancreatic adenocarcinoma cells (Panc-1), which display large morphological deformations to transit through 7 μm width channels and are unable to migrate across 3 μm widths, with only parts of the cytoplasm extending into the microchannels [22]. The results reported here are largely consistent with previous studies of migration of cells across microchannel structures in terms of impeding migration as a function of channel width, as we observed channel exclusion of primary myoblast cells, most pronounced in the 3 μm width channels with comparable height of the microchannel from previous reports (10–11 μm).

The incorporation of different geometric structures in the form of microfluidic microchannels i facilitates the study of how cell migration is affected under confinement. Prior studies have leveraged the availability of narrow channels (< 6μm) to allow estimation of microenvironments that are relevant in vivo [23]. The sub micrometer range of the extracellular space in native tissue is not permissive to fibroblast like cellular migration as there is no room for the formation of extensions of lamellipodium [31]. As is observed in our study, the highest percentage of cells are excluded when tasked with transmigration across the 3 μm channel or channel lengths of 500 μm. This may be due to extensive deformation and reorganization of the cytoskeletal architecture. To elucidate the cytoskeletal reorganization under confinement, Tong et al. (2012) demonstrated that HOS cells migrating through 3 μm-wide microchannels contact all microchannel walls, lose both leading and trailing edges and uniformly fill the volume of the microchannel. In our study, we readily observed this cellular morphology, as primary myoblasts underwent significant deformation, uniformly filled the microchannels, and became depolarized, which was most evident in the 500 μm length and 5 μm-wide microchannels (Figure 6a). The observed changes in cellular morphology is made possible by elongation of the nucleus, concentration of α—tubulin at the perinuclear region, and enrichment of filamentous actin at the poles of the cells [23]. A major role in impeding migration into microchannels and the corresponding speed has also been attributed to the compression of the nuclear region in the cell. Not only is the nucleus a large organelle (3–15 μm) [32], it is also 2–10 times stiffer than the cytoplasmic region [33]. Therefore, the combination of rigidity and large size may limit the cells to migrate in constricted channels which are less than 5 μm in diameter [32,34].

To further assess whether the observed physical constriction impeding myoblast migration can be overcome in the presence of a chemoattractive gradient, we investigated chemotactic migration of myoblasts under confinement in response to muscle-specific signaling growth factors such as HGF and bFGF. Both HGF and bFGF induced a significant increase in the number of successful crossings in the distal chamber at both wider microchannels (8–20 µm-wide channels) and smaller width microchannels (6.5–8 µm-wide channels). However, below 5 µm, no significant effect of growth factor treatment was observed. This suggests that below a critical width, physical constriction may influence migration behavior beyond growth factor driven chemoattraction. Previous studies corroborate our results as it has been demonstrated that various cell lines migrate in response to growth factors across PDMS-based microchannels and are influenced by the width of the microchannel and the concentration of the factor [23,35]. For example, Tong et al. demonstrated that HOS cells readily migrate across 10–20 µm-wide microchannels in response to 10% FBS and only a fraction of cells are able to exit the microchannels at channel widths below 6 µm [23]. Interestingly, in our study, we also observed a differential chemotactic effect between HGF and bFGF, where bFGF induced significantly greater myoblast migration. It has been demonstrated that human myoblast migration can be mediated through interactions with extracellular matrix and bFGF. Myoblasts have a higher migration velocity when cultured on laminin substrates and the presence of bFGF acts as an additive effect [18]. In our preparation, the microfluidic devices are functionalized with laminin to enhance cellular adhesion, and the observed increase in migration potential may be due to interactions between laminin and bFGF. Moreover, prior studies have demonstrated slight increase in migration in response to higher concentrations of HGF (100 ng/mL) [29,36,37]. Therefore, the concentration of applied HGF may play a role in migration and this has to be elucidated when cells migrate in confined environments.

In summary, these studies highlight the importance of microchannel dimensions in cellular migration over time scales which are relevant for cultivation and maturation of primary myoblasts. The use of microfluidic systems allows the incorporation of other regulatory factors of migration such as chemoattractants in the form of growth factors and addition of extracellular matrix proteins, which can further model the complex interactions cells undergo which may provide useful insight into migratory behavior, development, and morphogenesis.

## 5. Conclusions

The current study presents a systematic investigation of spontaneous primary myoblast migration across microchannels of different widths using microfluidic systems. The results demonstrate that myoblasts can be confined to a seeding well, which is interconnected via microchannels. The size of the microchannel in terms of available width and length affect the ability of myoblasts to successfully cross over to the adjacent chamber over time scales of 24 to 48 h. These findings can be used for the design of multicellular systems in co-culture designs.

## Figures and Tables

**Table 1:** Dimensions of the Central Ladder Channels Connecting the Proximal and Distal Chambers.

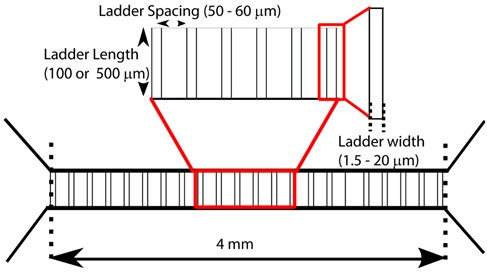					
Device Type	Ladder Width (µm)	Ladder Spacing (µm)	Ladder Height (µm)	Ladder Length (µm)	# of Parallel Channels
Variable Width*	1.5–20	60	10	100	6/width
3 µm channels	3	50	10	100	112
4 µm channels	4	50	10	100	109
5 µm channels	5	50	10	100	108
8 µm channels	8	50	10	100	102
5 µm channels	5	50	10	500	108
* channel widths: 6 channels/width: 1.5, 2.0, 3.0, 4.0, 5.0, 6.5, 8.0, 10.0, 15.0 & 20.0 µm
